# Retinoic Acid–Related Orphan Receptor α Is Required for Generation of Th2 Cells in Type 2 Pulmonary Inflammation

**DOI:** 10.4049/jimmunol.2200896

**Published:** 2023-06-30

**Authors:** Joseph Roberts, Anne Chevalier, Heike C. Hawerkamp, Aoife Yeow, Laura Matarazzo, Christian Schwartz, Emily Hams, Padraic G. Fallon

**Affiliations:** * School of Medicine, Trinity Biomedical Sciences Institute, Trinity College Dublin, Dublin, Ireland; † Mikrobiologisches Institut–Klinische Mikrobiologie, Immunologie und Hygiene, Universitätsklinikum Erlangen and Friedrich-Alexander Universität Erlangen-Nürnberg, Erlangen, Germany; ‡ Medical Immunology Campus Erlangen, Friedrich-Alexander Universität Erlangen-Nürnberg, Erlangen, Germany; § Trinity Translational Medicine Institute, Trinity College Dublin, Dublin, Ireland

## Abstract

The transcription factor retinoic acid–related orphan receptor α (RORα) is important in regulating several physiological functions, such as cellular development, circadian rhythm, metabolism, and immunity. In two in vivo animal models of type 2 lung inflammation, *Nippostrongylus brasiliensis* infection and house dust mite (HDM) sensitization, we show a role for *Rora* in Th2 cellular development during pulmonary inflammation. *N. brasiliensis* infection and HDM challenge induced an increase in frequency of *Rora*-expressing GATA3^+^CD4 T cells in the lung. Using staggerer mice, which have a ubiquitous deletion of functional RORα, we generated bone marrow chimera mice, and we observed a delayed worm expulsion and reduced frequency in the expansion of Th2 cells and innate lymphoid type 2 cells (ILC2s) in the lungs after *N. brasiliensis* infection. ILC2-deficient mouse (*Rora^fl/fl^Il7raCre*) also had delayed worm expulsion with associated reduced frequency of Th2 cells and ILC2s in the lungs after *N. brasiliensis* infection. To further define the role for *Rora*-expressing Th2 cells, we used a CD4-specific *Rora*-deficient mouse (*Rora^fl/fl^CD4Cre*), with significantly reduced frequency of lung Th2 cells, but not ILC2, after *N. brasiliensis* infection and HDM challenge. Interestingly, despite the reduction in pulmonary Th2 cells in *Rora^fl/fl^CD4Cre* mice, this did not impact the expulsion of *N. brasiliensis* after primary and secondary infection, or the generation of lung inflammation after HDM challenge. This study demonstrates a role for RORα in Th2 cellular development during pulmonary inflammation that could be relevant to the range of inflammatory diseases in which RORα is implicated.

## Key Points

- There is a role for *Rora* in Th2 cell development.
- *Rora* CD4-deficient mice expel worms and develop allergic lung inflammation.

## Introduction

The transcription factor retinoic acid-receptor related orphan receptor α (RORα) is known to regulate several physiological functions, such as cellular development, circadian rhythm, metabolism, and immunity (1). RORα is expressed in different immune cells populations with roles in innate lymphoid type 2 cells (ILC2) lineage commitment (2, 3), Th17 development (4), regulatory T cell (Treg) function (5), and macrophages (6, 7). There are several studies that now report *RORA*/*Rora* expression in Th2 cells (8–11), with *Rora* having a role in regulating activated Th cells during inflammation (11). Studies have shown that after helminth infection of mice, the transcriptional profile of Th2 cells and ILC2 in the lung are closely related relative to naive CD4 T cells from the lung and Th2 cells from the lymph nodes (12), with *Rora* transcripts in Th2 cells correlated with activated and cytokine-secreting cells (11). Another recent study demonstrated that RORα repressed T cell development but promoted ILC2 development in the thymus (13). A complete understanding of the role of RORα in the generation and function of Th2 cells in the context of lungs inflammation remains elusive.

To investigate the role of RORα in the in vivo development of pulmonary type 2 responses, we used *Rora*–yellow fluorescent protein (YFP) reporter mice and mice with conditional deficiency of *Rora* in ILC2 and CD4 cells. Mice were subjected to two distinct pulmonary type 2 inflammatory models: *Nippostrongylus brasiliensis* infection and house dust mite (HDM) sensitization. We identified a population of *Rora*-expressing CD4 T cells that coexpressed the Th2 cell master transcription factor GATA3^+^ cells in the lung. After *N. brasiliensis* infection and HDM challenge, we observed an increase in frequency of *Rora*-expressing CD4 T cells expressing integrin αEβ7 (CD103), a cell marker of cells that resides within the epithelium of mucosal organs (14). Further to this, we generated *Rora*^sg/sg^ bone marrow (BM) chimera (BMC) mice from *Rora*^sg/sg^ mice, which have a ubiquitous deletion of functional RORα, to explore the role of *Rora* in cells from hematopoietic versus nonhematopoietic origin. In addition to the known deficiency in ILC2s and delayed expulsion of worms (2), it was also observed that *Rora*^sg/sg^ BMC mice had a reduced frequency in the expansion of GATA3^+^CD4 T cells in the lungs after *N. brasiliensis* infection. In support of these observations, we used another ILC2-deficient mouse (*Rora^fl/fl^Il7raCre*), which had a delayed worm expulsion and reduced frequency of lung ILC2s, as reported by Oliphant et al. (15), and diminished frequency of Th2 in the lungs after *N. brasiliensis* infection. CD4-specific *Rora*-deficient (*Rora^fl/fl^CD4Cre*) mice had significantly reduced frequency of lung Th2 cells after *N. brasiliensis* infection and HDM challenge. However, the reduction in pulmonary Th2 cells in *Rora^fl/fl^CD4Cre* mice did not impact the expulsion of *N. brasiliensis* after primary and secondary infection, or alter the generation of lung inflammation after HDM sensitization. Therefore, this study highlights the importance of *Rora* in GATA3^+^CD4^+^ T cell development in the lungs during type 2 pulmonary inflammation.

## Materials and Methods

### Animals

C57BL/6J (wild type [WT]), staggerer *Rora* spontaneous mutant (JAX strain number 002651; *Rora*^sg/sg^), B6.129X1-*Gt(ROSA)26Sortm1(EYFP)Cos*/J (JAX strain number 006148), *B6.SJL-Ptprc^a^Pepc^b^/Boyj* (JAX strain number 002014; CD45.1^+^), *Tg(Cd4-cre)1Cwi/BfluJ* (JAX strain number 017336; *CD4^Cre^*), *Tg(Cd4-Cre/ERT2)11Gnri/j* (JAX strain number 022356; *CD4^CreERT2^*), and *Id2^tm1.1(Cre/ERT2)Blh/^ZhuJ* (JAX 016222; *ID2^CreERT2^*) mice were purchased from Jackson Laboratories (Bar Harbor, MD). *Il7r^tm1.1(icre)Hrr^* (*IL7ra^Cre^*) mice were as described in Schlenner et al. (16). *Rora^tm1(cre)Ddmo^* mice (17) were crossed with R26R-EYFP mice to generate *Rora*-YFP reporter mice (referred to herein as *Rora*-YFP). Conditional *Rora* floxed mice were generated (Lexicon Pharmaceuticals), and homozygous mice were crossed with *CD4^Cre^*, *Il7raCre*, *ID2CreERT2*, and *CD4CreERT2* mice to generate *Rora^fl/fl^CD4^Cre^*, *Rora^fl/fl^Il7ra^Cre^* (15), *Rora^fl/fl^ID2^CreERT2^*, and *Rora^fl/fl^CD4^CreERT2^* animals, with a conditional deletion of *Rora* in CD4 and IL-7Rα–expressing cells and tamoxifen-inducible deletion of *Rora* in CD4 and ID2-expressing cells, respectively. All groups of experimental mice were matched for age, and female mice were used in all experiments. Animals were housed in a specific pathogen-free facility in individually ventilated and filtered cages under positive pressure. It is relevant that due to the importance of RORα in regulating circadian rhythm (18), for all in vivo experiments, mice were sensitized, infected, and killed between 7 and 10 am to avoid variations related to alterations in the circadian rhythm because of time differences in experiments. All experiments were performed in compliance with Ireland’s Health Product’s Regulatory Authority and approved by the Trinity College Dublin’s Animal Research Ethics Committee.

### BMC generation

BMC mice were generated as previously described (7). CD45.1^+^C57BL/6 mice were used as recipient mice and were reconstituted with BM from WT or *Rora*^sg/sg^ mice. *Rora*^sg/sg^ mice could not be used as recipient mice to generate BMC because they would not survive irradiation and have stunted growth. In brief, CD45.1^+^C57BL/6 recipient mice were irradiated using an X-ray irradiator (XStrahl CIX3), receiving 9 Gy in two doses (5 and 4 Gy) 3 h apart. Mice were then reconstituted with 1 × 10^7^ BM cells isolated from either CD45.2^+^C57BL/6 mice or *Rora*^sg/sg^ mice. BM reconstitution efficiency was assessed by flow cytometry analysis of peripheral blood from mice before being infected with *N. brasiliensis*.

### N. brasiliensis *infection*

*N. brasiliensis* is maintained by passage through female Wistar rats. Mice were s.c. injected with 500 live infective larval stage 3 *N. brasiliensis*. Lungs were taken from infected mice at the indicated time points postinfection, as well as from uninfected mice. The small intestines of infected mice were removed, and adult worm numbers were enumerated using a dissecting microscope.

### Tamoxifen treatment

*Rora^fl/fl^*, *Rora^fl/fl^ID2^CreERT2^*, and *Rora^fl/fl^CD4^CreERT2^* mice were injected three times (days −9, −8, and −7 preinfection) i.p. with 75 mg/kg body weight tamoxifen (Sigma-Aldrich) dissolved in oil. Mice were rested for 1 wk after the final injection before they were infected with *N. brasiliensis.*

### House dust mite

HDM extracts (*Dermatophagoides pteronyssinus*) were purchased from Stallergenes Greer (Derp1 146.45 mcg/vial, protein 2.26 mg/vial, and endotoxin 812.5 endotoxin units/vial). Mice were challenged with HDM to induce allergic airway inflammation, as described by Plantinga et al. (19). In brief, mice were sensitized with 1 μg HDM in 20 μl PBS, via intranasal (i.n.) injection. The mice were then challenged for 5 consecutive days from day 7 to day 11 with 10 μg HDM in 20 μl PBS via i.n. administration. At day 14, mice were sacrificed and analyzed. Control mice were sensitized and challenged i.n. with PBS.

### Cell isolation

Lungs were isolated as previously described (20). In brief, lungs were minced and incubated in collagenase D (1 mg/ml; Roche, Dublin, Ireland) for 30 min at 37°C. Single-cell suspensions were then obtained from lungs by filtering through a 70-μm cell strainer (Falcon, Corning). RBC contamination was removed by incubation with RBC lysis buffer (BD Pharm lyse). Cells were analyzed by flow cytometry. In some experiments, the murine lungs were removed for assessment of tissue cytokines (IL-4, IL-5, IL-17, and IFN-γ) or histological analysis.

### Flow cytometry

Cells were stained with BD Biosciences (Oxford, U.K.) mAbs: CD45-PerCP-Cy5.5 (30-F11), CD45.2-V450 (104), CD4-BV650 (RM4-5), CD3-FITC (17A2), CD11b-eFluor450 (M1/70), CD19-BV711 (1D3), SiglecF (E50-2440), CD103-PE-CF594 (M290), Ly6G-BV650 (1A8); eBioscience (Loughborough, U.K.) mAbs: CD4-allophycocyanin-eFluor780 (RM4-5); Invitrogen (Dublin, Ireland) mAbs: KLRG1-PE-eFluor610 (2F1) and CD127-PerCP-ef710 (SB/199); and BioLegend (London, U.K.) mAbs: CD45-BV711 (clone: 30-F11), CD3-BV605 (17A2), CD11b-allophycocyanin-Cy7 (M1/70), CD11c-PE-Cy7 (N418), Ly6G-BV785 (1A8), Ly6C-BV606 (HK1.4), and SiglecF-allophycocyanin (S1700L). Before surface staining, Fc receptors were blocked using Fc-Block CD16/32 (BD Biosciences), and cells were incubated with LIVE/DEAD Fixable Aqua stain (Molecular Probes, Invitrogen) to isolate dead cells. For staining of transcription factors, cells were fixed and permeabilized using the Foxp3 staining buffer kit (Invitrogen) and stained with mAbs: GATA3-PE (TWAJ) and Foxp3-PE-Cy7 (FJK-16s). For the detection of YFP, along with intracellular transcription factors from *Rora*-YFP mice, after surface markers and viability stain, cells were prefixed with 2% paraformaldehyde followed by Foxp3 staining buffer kit. Cells were analyzed using a BD Fortessa (BD Biosciences), and data were analyzed using FlowJo software (Tree Star, Ashland, OR), using appropriate controls.

### Cell sorting

*Rora-*YFP–expressing CD4 T cells were isolated from splenocytes of *Rora* reporter mice using the BD FACS Aria Fusion. Cells were identified as CD45^+^CD4^+^YFP^+^ or CD45^+^CD4^+^YFP^−^. The gating strategy for CD45^+^CD4^+^YFP^+/−^ cell sorting and analysis of purity are provided in Supplemental Fig. 1. For in vitro studies, naive CD4 T cells were isolated from murine spleens by MACS, as per the manufacturer’s instructions (Miltenyi Biotec). Cells were labeled with biotin–Ab mixture containing Abs conjugated against CD8a, CD11b, CD11c, CD19, CD45R (B220), CD49b (DX5), CD105, Anti-MHCII, Ter-119, and TCRγ in MACS buffer according to the manufacturer’s instructions and separated using an AutoMACS system (Miltenyi Biotec).

### In vitro CD4 T cell polarization

Naive CD4 T cells were isolated from spleens and polarized into Th2 cells, as described in Schwartz et al. (20). Naive CD4 T cells were cultured for 5 d with plate-bound anti-CD3 (2 μg/ml; clone: 145-2C11; BD Biosciences), soluble CD28 (2 μg/ml; clone: 37.51; BD Biosciences). Cells were cultured in nonpolarizing Th0 conditions, IL-2 (20 ng/ml), or Th2 polarization conditions, IL-2 (20 ng/ml) and IL-4 (20 ng/ml; R&D Systems, Abingdon, U.K.).

### RNA isolation and real-time PCR

RNA was isolated from FACS-sorted cells using RNeasy kit and reverse transcribed using the QuantiTect Reverse Transcription Kit incorporating a genomic DNA elimination step (Qiagen, Germantown, MD), as previously described by Hams et al. (6). Real-time quantitative PCR was performed on an ABI Prism 7900HT sequence detection system (Applied Biosystems, Dublin, Ireland) using predesigned TaqMan gene expression assays specific for murine *Rora* (Mm004431303_m1) and normalized to murine 18s. Relative fold expression was calculated using the ΔΔ*C_t_* method of analysis.

### Bronchoalveolar lavage

Bronchoalveolar lavage (BAL) fluid was collected from HDM-challenged and naive mice, as described previously (21). The total and differential cell counts were performed on BAL cells after cytospin and Diff-Quik staining.

### Lung cytokine quantification by ELISA

Cytokine levels were quantified in murine lung homogenates by sandwich ELISA. Lungs were homogenized as described previously (22). All cytokines (IL-4, IL-5, IL-17, and IFN-γ) were measured with the DuoSet ELISA development system from R&D Systems (Abingdon, U.K.) following the manufacturer’s protocol. Cytokine levels were normalized to total lung protein after BCA reagent assay.

### Histological analysis

Lungs from mice challenged with HDM or PBS were fixed in 10% formaldehyde saline, followed by paraffin embedding. Sections of 4 μm were cut, and then slides were stained with H&E or periodic acid–Schiff (PAS). PAS-stained goblet cells in airway epithelium were quantified using a numerical scoring system (0: <5% goblet cells; 1: 5–25%; 2: 25–50%; 3: 50–75%; 4: >75%), as described by Mangan et al. (22). Histology images were acquired using an Aperio ScanScope at 20× original magnification and analyzed using Aperio ImageScope software.

### Statistics

Statistical analysis was performed using Prism 8 (GraphPad Software). Results are presented as mean ± SEM. Statistical differences between groups were analyzed by ANOVA, unpaired Student *t* test, or by two-tailed Mann–Whitney *U* test. The *p* values were considered significant when *p < 0.05, **p < 0.01, ***p < 0.001, and ****p < 0.0001.

### Ethics statement

The animal study was reviewed and approved by Trinity College Dublin’s Animal Research Ethics Committee.

## Results

### Expansion of *Rora*-expressing CD4 T and Th2 cells in lungs after *N. brasiliensis* infection

**FIGURE 1.**
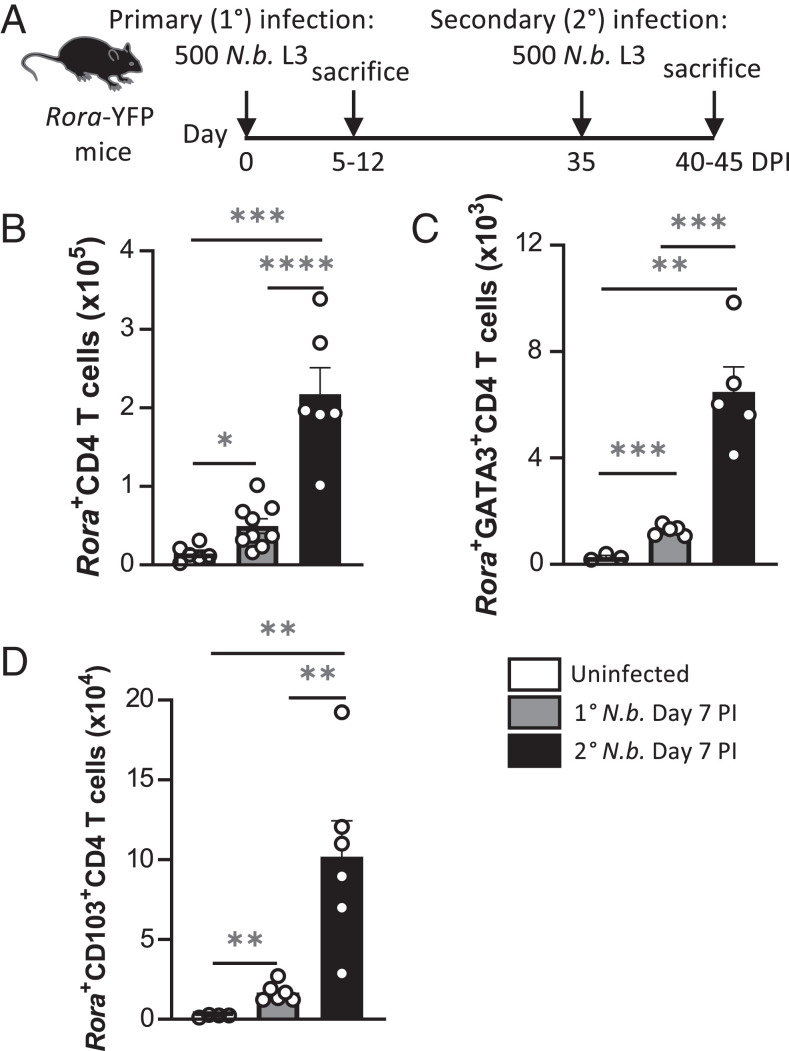
Identification and expansion of lung *Rora-*expressing CD4 T cells after *N. brasiliensis* infections. Lungs were isolated from *N. brasiliensis*–infected *Rora* reporter mice and assessed by flow cytometry at day 7 after primary and secondary infection. (**A**) Schematic diagram of the experimental design of *N. brasiliensis* infections. (**B**) *Rora-*expressing CD4 T cells in the lungs after *N. brasiliensis* infection (*n* = 6–7). (**C**) *Rora-*expressing GATA3^+^CD4 (Th2) T cells in the lungs after *N. brasiliensis* infection (*n* = 3–5). (**D**) *Rora-*expressing CD103^+^CD4 T cells in the lungs after *N. brasiliensis* infection (*n* = 4–6). Data are representative of three separate experiments and are presented as mean ± SEM. Student *t* test was used for statistical analysis: *p < 0.05, **p < 0.01, ***p < 0.001, ****p < 0.0001. DPI, day postinfection; *N.b.*, *N. brasiliensis*; ns, not significant.

To assess *Rora*-expressing immune cells in the lung during type 2 inflammation, we infected *Rora* reporter mice, which express a YFP in *Rora*-expressing cells, with the helminth *N. brasiliensis* (Fig. 1A). Flow cytometry and *t*-distributed stochastic neighbor embedding clustering analyses identified *Rora*-expressing CD45^+^ cells, including a population of *Rora*-expressing ILC2 and CD4^+^ T cells in lungs of mice 7 d after primary infection (Supplemental Fig. 2). *Rora* reporter mice received a primary *N. brasiliensis* infection, followed by a secondary infection 35 d after the first infection, to further investigate *Rora*-expressing CD4^+^ cells in the lungs (Fig. 1A). There was a significant (*p* < 0.05) increase in frequency of *Rora*-expressing CD4^+^ T cells in lung of primary infected mice, compared with uninfected mice, with a further significant (*p* < 0.001) increase after secondary infection (Fig. 1B). This is in support with other studies that reported an increase in lung CD4 T cells expressing *Rora* after infection and allergens, including ragweed pollen, papain, and OVA (11, 23).

There was an increase in *Rora*^+^CD4 T cells and CD45^+^CD3^+^CD4^+^GATA3^+^ cells (Supplemental Fig. 3A) in the lungs of mice postinfection, with the highest frequency in cells during a secondary infection (Fig. 1C). Furthermore, there was a significant (*p* < 0.01) increase in *Rora*-expressing CD4 T cells expressing a tissue-resident marker, CD103 (CD4^+^CD103^+^*Rora*-YFP^+^; Supplemental Fig. 3B), after helminth infection (Fig. 1D). These data identify an increase in a population of *Rora*-expressing GATA3^+^CD4 Th2 cells in the lungs of mice after primary and secondary helminth infection.

### Reduction in lung GATA3^+^CD4 cells in *Rora-*deficient chimera mice

**FIGURE 2.**
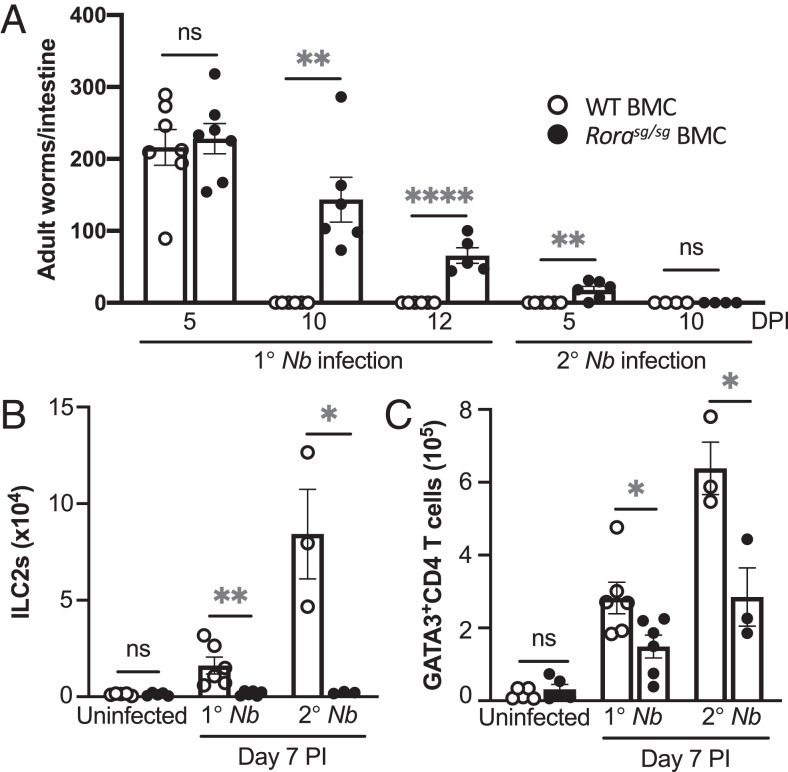
Delayed worm rejection and impaired generation of ILC2s and Th2 cells in *N. brasiliensis*–infected *Rora*-deficient mice. (**A**) Increase in worms in small intestine of *Rora^sg/sg^* BMC mice on days 10 and 12 after primary (1°) and day 5 after secondary (2°) *N. brasiliensis* infection compared with WT BMC mice (*n* = 4–7). (**B** and **C**) Numbers of ILC2s (B) and GATA3^+^CD4 T (Th2) cells (C) in lungs of *Rora^sg/sg^* BMC and WT BMC mice after *N. brasiliensis* infection (*n* = 3–6). Data are representative of three separate experiments and are shown as mean ± SEM. Student *t* test was used for statistical analysis: *p < 0.05, **p < 0.01, ****p < 0.0001. DPI, day postinfection; *Nb*, *N. brasiliensis*; ns, not significant.

To further explore the roles for RORα in immune cells during helminth infection, we used *Rora^sg/sg^* mutant mice, which produce a ubiquitously expressed, truncated form of the RORα protein to generate *Rora^sg/sg^* BMC mice. *Rora^sg/sg^* BMC and WT BMC mice were infected with *N. brasiliensis*. In WT BMC mice, worms were recovered from the small intestines on day 5 after primary infection but were expelled by day 10, and these mice were resistant to secondary infection (Fig. 2A). In contrast, *Rora^sg/sg^* BMC mice had a delayed worm expulsion after primary infection, and worms were present in the small intestines after secondary *N. brasiliensis* infection (Fig. 2A). Although there was an increase in lung ILC2s (CD45^+^CD3^−^CD4^−^SiglecF^−^CD11b^−^CD127^+^KLRG1^+^GATA3^+^; Supplemental Fig. 3A) after *N. brasiliensis* infection in WT BMC mice (Fig. 2B), Rorasg*^/sg^* BMC mice did not have an increase in the number of lungs ILC2s and had significantly (*p* < 0.01 and *p* < 0.5, respectively) fewer ILC2s compared with WT BMC after primary and secondary helminth infection (Fig. 2B). This agrees with *Rora^sg/sg^* BMC mice having an impaired development of ILC2 with delayed worm expulsion after helminth infection (2). Interestingly, *Rora^sg/sg^* BMC mice had significantly (*p* < 0.05) reduced frequency of GATA3^+^CD4 Th2 cells in the lungs compared with WT BMC mice, after both primary and secondary *N. brasiliensis* infection (Fig. 2C). These data demonstrate that *Rora*-deficient chimera mice have an altered type 2 immune response after *N. brasiliensis* infection characterized by reduced frequency of GATA3^+^CD4 Th2 cells and ILC2s in the lungs and an associated delayed worm expulsion.

### Impaired generation of GATA3^+^CD4 cells in the lungs of *Rora*^fl/fl^IL7raCre mice after helminth infection

**FIGURE 3.**
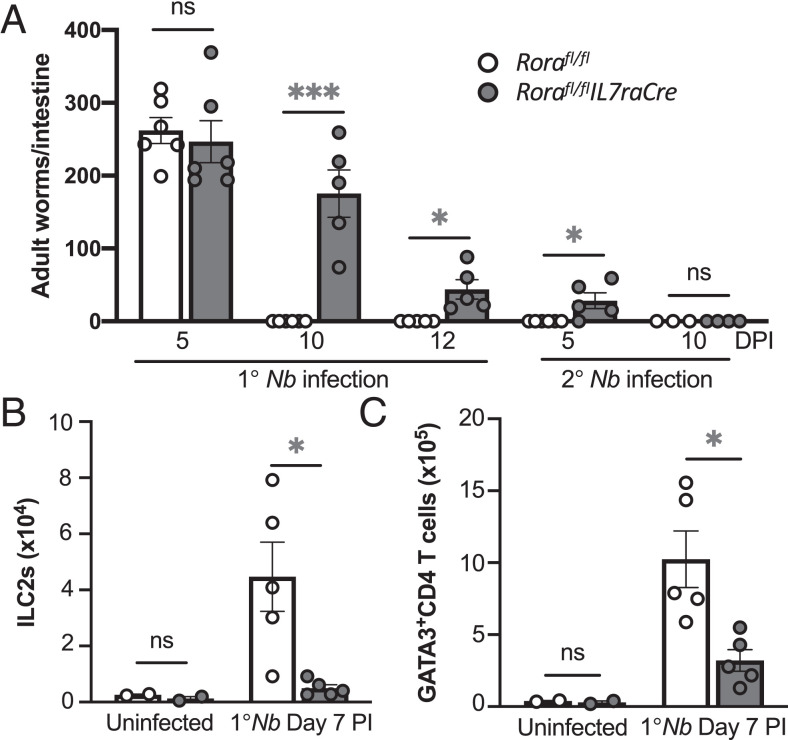
*Rora^fl/fl^Il7raCre* mice have delayed worm rejection with impaired generation of ILC2s and Th2 cells. (**A**) Worm expulsion in *Rora^fl/fl^Il7raCre* and *Rora^fl/fl^* mice after primary (1°) and secondary (2°) *N. brasiliensis* infection (*n* = 2–6). (**B** and **C**) Reduced frequency of ILC2s (B) and GATA3^+^CD4 T (Th2) cells (C) in the lungs of Rorafl*^/fl^Il7raCre* mice at day 7 after 1° *N. brasiliensis* infection, compared with WT mice (*n* = 2–5). Data are representative of three separate experiments and shown as mean ± SEM. Student *t* test was used for statistical analysis. *p < 0.05, ***p < 0.001. DPI, day postinfection; *Nb*, *N. brasiliensis*; ns, not significant.

To further address the role of *Rora*-expressing cell in the lungs after nematode infection, we used *Rora^fl/fl^IL7raCre* mice, in which have *Rora* is excised from *Il7ra*-expressing cells, which has previously been identified as an ILC2-deficient mouse strain (15). Similar to what was reported by Oliphant et al. (15), *Rora^fl/fl^IL7raCre* mice had a delayed worm expulsion after primary *N. brasiliensis* infection **(**Fig. 3A). Interestingly, after secondary *N. brasiliensis* infection, worms were detected in *Rora^fl/fl^IL7raCre* mouse small intestines, thus indicating an impaired development of a functional type 2 immune response in *Rora^fl/fl^IL7raCre* mice (Fig. 3A). Assessment of the cellular response in the lungs showed, as anticipated, that *Rora^fl/fl^IL7raCre* mice have significantly (*p* < 0.05) reduced frequency of lung ILC2s after helminth infection (Fig. 3B). In addition, *Rora^fl/fl^IL7raCre* mice also had a significantly (*p* < 0.05) reduced frequency of lung GATA3^+^CD4 Th2 cells after helminth infection, compared with infected *Rora^fl/fl^* mice (Fig. 3C). These data indicate that the impaired generation of a functional type 2 immune response in *Rora^fl/fl^IL7raCre* mice after *N. brasiliensis* infection may be caused by associated diminished capacity to generate GATA3^+^CD4 Th2 cells in the lungs, as well as a reported defect in ILC2s.

### *Rora-*expressing CD4 T cells are not required for *N. brasiliensis* worm expulsion

**FIGURE 4.**
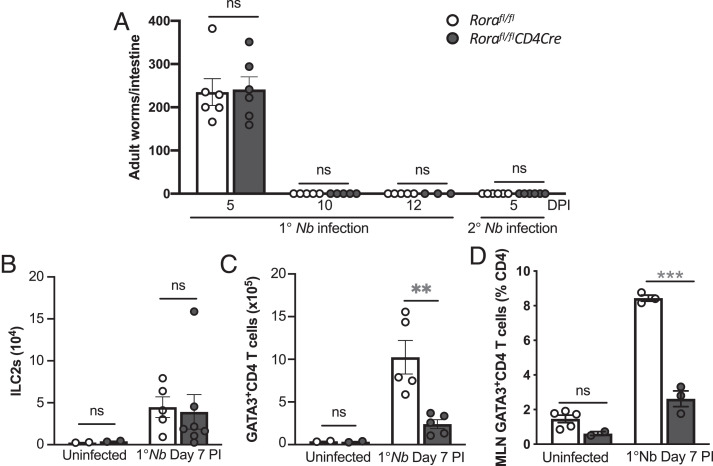
*Rora*-expressing CD4 cells are redundant in type 2–mediated expulsion of *N. brasiliensis* infection. (**A**) Comparable worm expulsion in primary (1°) and secondary (2°) *N. brasiliensis* infection between *Rora^fl/fl^CD4Cre* mice and control *Rora^fl/fl^* mice (*n* = 3–5). (**B** and **C**) Comparable frequency of lung ILC2s (B) and GATA3^+^CD4 T (Th2) cells (C) in *Rora^fl/fl^CD4Cre* mice compared with control mice, at day 7 after 1° *N. brasiliensis* infection (*n* = 2–7). (**D**) Frequency of GATA3^+^CD4 T cells in the mesenteric lymph nodes (MLNs) of *Rora^fl/fl^CD4Cre* mice and *Rora^fl/fl^* mice after primary (1°) *N. brasiliensis* (*n* = 2–5). Data are representative of three separate experiments and shown as mean ± SEM. Student *t* test was used for statistical analysis: **p < 0.01, ***p < 0.001. DPI, day postinfection; *Nb*, *N. brasiliensis*; ns, not significant.

We have identified that after *N. brasiliensis* infection there are *Rora*-expressing GATA3^+^CD4 Th2 cells in the lungs, and in two distinct *Rora*-deficient mouse strains (*Rora^sg/sg^* BMC and *Rora^fl/fl^Il7raCre* mice) there is impaired generation of pulmonary GATA3^+^CD4 Th2 cells. Therefore, to further define the role of RORα in CD4 cells, we used *Rora^fl/fl^CD4Cre* mice, where *Rora* gene is excised in cells expressing the *CD4* gene. After primary and secondary infection with *N. brasiliensis*, *Rora^fl/fl^CD4Cre* mice had comparable worm expulsion with *Rora^fl/fl^* mice (Fig. 4A). Assessment of the lung cellular immune response revealed that there was no significant difference in frequency of ILC2s in *Rora^fl/fl^CD4Cre* and WT mice, in uninfected mice, and after *N. brasiliensis* infection (Fig. 4B). However, *Rora^fl/fl^CD4Cre* mice had a significantly (*p* < 0.01) reduced frequency of lung GATA3^+^CD4 T cells, compared with WT mice after *N. brasiliensis* infection (Fig. 4C). *N. brasiliensis*–infected *Rora^fl/fl^CD4Cre* mice also had reduced frequency of GATA3^+^CD4 T cells in the mesenteric lymph node compared with *Rora^fl/fl^* mice (Fig. 4D), supporting the role of *Rora* in the in vivo expansion of Th2 cells. To further address the role of *Rora* in the development of Th2 cells, we isolated CD4 T cells from the spleen of WT or *Rora*-deficient mice and cultured them in vitro under Th2 cell polarization conditions. Naive CD4 T cells from *Rora^fl/fl^CD4Cre* mice had significantly reduced (*p* < 0.01) capacity to expand to GATA3^+^ Th2 cells after in vitro Th2 polarization compared with the generation of Th2 cells from CD4^+^ cells from WT *Rora^flfl^* mice (Supplemental Fig. 4A, 4B). Therefore, these results indicate that although RORα expression in CD4 cells is required for both in vitro and in vivo Th2 cellular development, lung *Rora*-expressing CD4^+^ Th2 cells are not required to mediate worm expulsion after primary and secondary *N. brasiliensis* infection.

### *Rora* GATA3^+^CD4^+^ cells in lungs of allergen-sensitized mice do not contribute to lung inflammation

**FIGURE 5.**
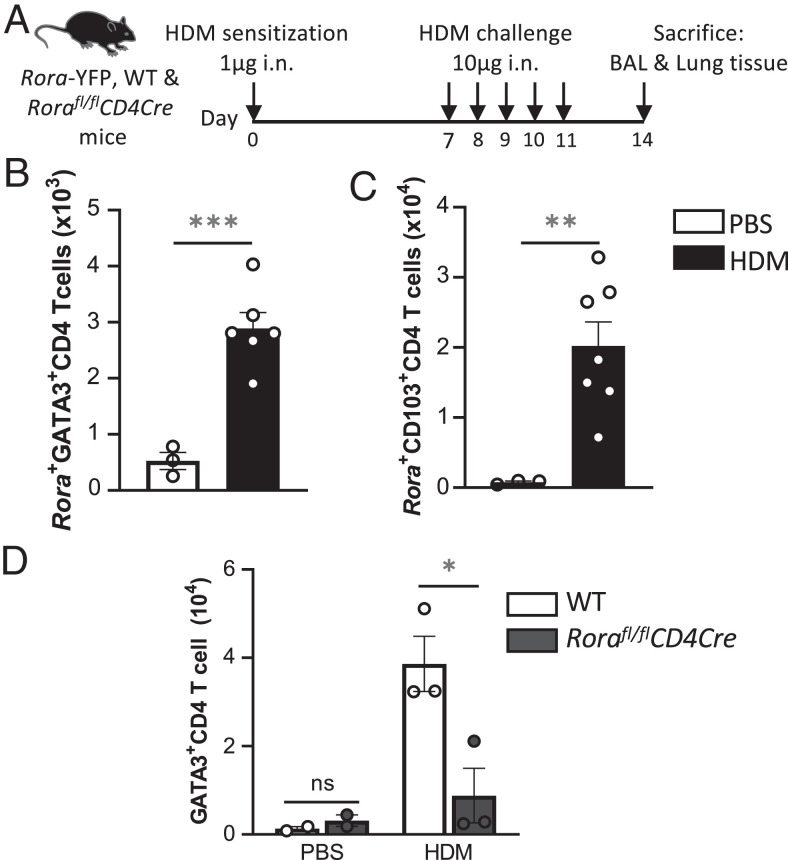
Identification of *Rora-*expressing Th2 cells in the lungs after HDM sensitization. (**A**) Schematic diagram of HDM-induced airway inflammation model. Mice were sensitized with 1 μg HDM i.n. at day 0 and challenged five times with 10 μg HDM at days 7–11. At day 14, BAL fluid and lung tissues were collected. Control mice received PBS at sensitization and challenges. (**B**) Numbers of *Rora-*expressing GATA3^+^CD4 T (Th2) cells in lungs of *Rora* reporter mice after HDM (*n* = 3–6). (**C**) Numbers of CD103^+^ *Rora*-expressing CD4 T cells in lungs of *Rora* reporter mice after HDM challenges (*n* = 3–7). (**D**) Numbers of GATA3^+^CD4 T (Th2) cells in lungs of *Rora^fl/fl^CD4Cre* mice after HDM, compared with WT mice (*n* = 2–3). Data are representative of three separate experiments and presented as mean ± SEM. Student *t* test was used for statistical analysis: *p < 0.05, **p < 0.01, ***p < 0.001. ns, not significant.

To expand on the findings generated using a helminth model of type 2 immunity, we used HDM as an allergen to induce lung inflammation (Fig. 5A). *Rora* reporter mice were i.n. sensitized and challenged with either HDM or PBS. In HDM-sensitized mice, there was a significant (*p* < 0.001) increase in frequency of *Rora* GATA3^+^CD4^+^ cells (Fig. 5B). In addition, there was a significant (*p* < 0.01) increase in *Rora*^+^CD103^+^CD4 T cells in the lungs after HDM sensitization (Fig. 5C). To further assess the role of RORα in lung Th2 cellular development, we sensitized *Rora^fl/fl^CD4Cre* mice with HDM. *Rora^fl/fl^CD4Cre* mice had a significantly (*p* < 0.05) reduced frequency of GATA3^+^CD4 T cells in the lungs after HDM, compared with sensitized WT mice (Fig. 5D).

**FIGURE 6.**
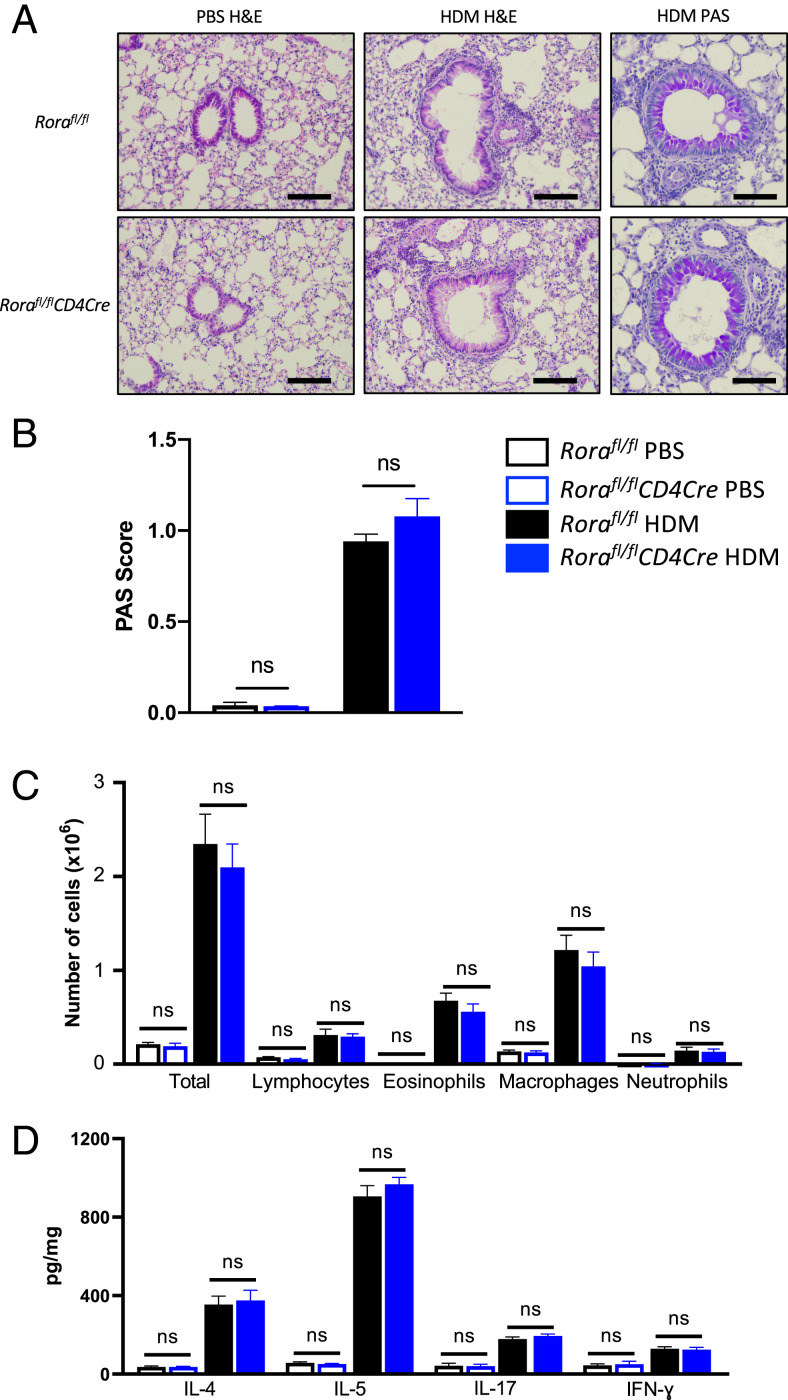
*Rora*-expressing Th2 cells are not required to generate allergic lung inflammation in mice after HDM sensitization. (**A**) Representative images of H&E- and PAS-stained lungs. Scale bar for H&E images, 50 μm. Scale bar for PAS images, 20 μm. (**B**) PAS score of lungs (*n* = 8–11). (**C**) Quantification of immune cells in BAL from *Rora*^fl/fl^ and *Rora^fl/fl^CD4Cre* mice after vehicle and HDM treatments (*n* = 3–7). (**D**) Levels of IL-4, IL-5, IL-17, and IFN-γ in lungs expressed as pg/mg lung protein. Data are from two separate experiments and are presented as mean ± SEM. Student *t* test was used for statistical analysis. ns, not significant.

Lung histological analysis revealed there was no significant difference in inflammation or goblet cell hyperplasia after HDM in *Rora^fl/fl^* and *Rora^fl/fl^CD4Cre* mice (Fig. 6A, 6B). In HDM-sensitized mice, there is an elevated number of immune cells (total cells, lymphocytes, eosinophils, macrophages, and neutrophils) in the BAL. However, there was no significant difference in the number of cells in BAL counts between *Rora^fl/fl^* and *Rora^fl/fl^CD4Cre* (Fig. 6C). Furthermore, despite the reduced frequency of lung Th2 cells in *Rora^fl/fl^CD4Cre* mice, there was no significant difference in cytokines (IL-4, IL-5, IL-10, IFN-γ) in the lung homogenate (Fig. 6D). These findings indicate that although the expression of *Rora* in CD4 T cells is involved in the expansion of GATA3^+^CD4 T cells in the lungs of HDM allergen–sensitized mice, deficiency of *Rora* in CD4 cells does not reduce the generation of allergen-induced type 2 pulmonary inflammation.

### *Rora*-expressing ILC2s cells are required for helminth expulsion

**FIGURE 7.**
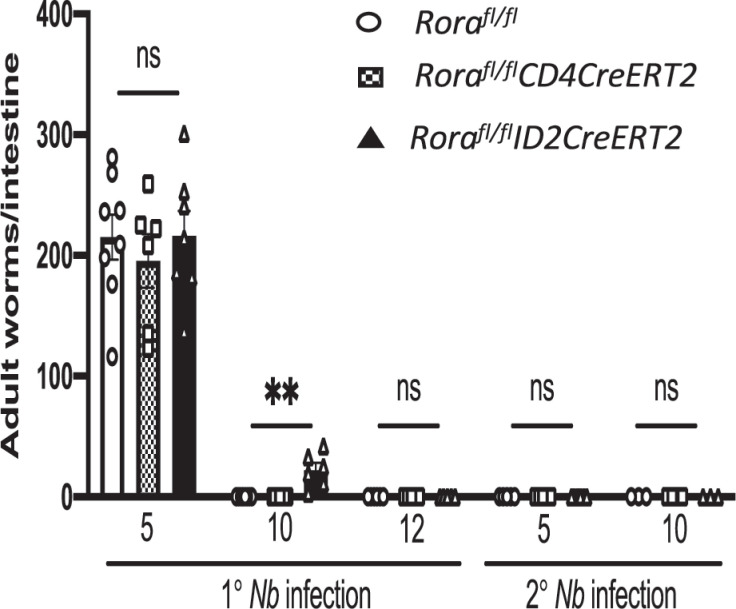
*Rora*-expressing ILC2s are required for helminth expulsion. Worm expulsion in *Rora^fl/fl^ID2CreERT2*, *Rora^fl/fl^CD4CreERT2*, and *Rora^fl/fl^* mice after primary (1°) and secondary (2°) *N. brasiliensis* infection (*n* = 3–8). Data are representative of mean ± SEM. Differences indicated as *p* values, as assessed by Student *t* test. **p < 0.01. *Nb*, *N. brasiliensis*; ns, not significant.

To further explore the relative roles of RORα cell-intrinsic expression in CD4 cells or ILC2 in the expulsion of worms after nematode infection, we used *CreERT2* mice for tamoxifen-inducible *Rora* deficiency in CD4 cells (*Rora^fl/fl^CD4CreERT2*) and ILC2s (*Rora^fl/fl^ID2CreERT2*). *Rora^fl/fl^CD4CreERT2* mice had comparable worm expulsion as control mice after nematode infection, whereas *Rora^fl/fl^ID2CreERT2* mice had delayed worm rejection after primary infection (Fig. 7). These data indicate that RORα deficiency in ILC2s impacts worm expulsion after primary helminth infection, although deficiency of RORα in CD4 cells is not required for the generation of the associated gut-mediated expulsion of worms.

## Discussion

Studies have shown that the transcription factor RORα has roles in cellular development, circadian rhythm, inflammation, and metabolism. We identify a role for RORα in Th2 (GATA3^+^CD4 T cells) cellular development in the lungs during type 2 pulmonary inflammation. We also identified a population of lung-resident *Rora*-expressing CD4 T cells, which expands after *N. brasiliensis* infection and HDM challenge. Indeed, Th2 cells are known to express *RORA*/*Rora* (8–11). However, the functional role of these lung-resident *Rora*-expressing Th2 cells remains undetermined.

As previously reported by Wong et al. (2), *Rora^sg/sg^* BMC mice were ILC2 deficient and have delayed *N. brasiliensis* expulsion after primary infection. In another ILC2-deficient mouse strain, *Rora^fl/fl^Il7raCre* mice, there was also delayed worm rejection after primary worm expulsion (15). We now demonstrate that in both *Rora*-deficient models, the delay in worm rejection is associated with reduced frequency of Th2 cells in the lungs of mice after primary and secondary helminth infection. Furthermore, we report that both strains have defective generation of the functional type 2 response that mediates resistance to secondary *N. brasiliensis* infection. In both ILC2-deficient strains, the roles of RORα on ILC2 versus Th2 cell functions are not defined. Regarding *Rora^fl/fl^Il7raCre* mice, Il7rα (CD127) is broadly expressed throughout the lymphoid system, with both ILC2s and CD4 T cells expressing CD127 (16, 24–26). Therefore, given the known communication between ILC2s and Th2 cells (15, 20, 27–29), the underlying mechanisms of RORα in ILC2 and Th2 cells remain unclear. Further studies are required to confirm whether the impact on CD4 T cells in *Rora^fl/fl^Il7raCre* mice is solely due to the ILC2 deficiency, or if there is a standalone effect of *Rora* deletion in Il7ra-expressing CD4 T cells.

To specifically address the impact of *Rora* in expressing CD4 cells, we used *Rora^fl/fl^CD4Cre* mice, which have *Rora* specifically deleted from *CD4*-expressing cells. We show that after primary and secondary *N. brasiliensis* infection, there was comparable worm counts between *Rora^fl/fl^CD4Cre* and *Rora^fl/fl^* mice. In addition, we report comparable frequency of ILC2s within lungs of *Rora^fl/fl^CD4Cre* and control mice, in both uninfected mice and after infection state. Furthermore, the frequency of Th2 cells in uninfected *Rora^fl/fl^* and *Rora^fl/fl^CD4Cre* mice was comparable, as reported by Haim-Vilmovsky et al. (11). However, *Rora^fl/fl^CD4Cre* mice have a reduced frequency of Th2 cells in the lungs and mesenteric lymph nodes after *N. brasiliensis* infection. The reduced frequency of Th2 cells in the lungs of *Rora^fl/fl^CD4Cre* mice is ILC2 independent. Furthermore, RORα CD4 cell deficiency affects Th2 development under in vitro Th2 cell polarization conditions, suggesting that RORα has a cell-intrinsic role in CD4 cells and the development of Th2 cells. However, although RORα expression in CD4 cells impacts on Th2 cell development, RORα deficiency in CD4 cells is not required for the associated expulsion of worms in the intestines. In contrast, RORα expression in ILC2s is required for the generation of immune responses to expel worms.

Previous studies have shown that *Rora* regulates Th cells during inflammation (11), although another study reported that RORα represses T cell development and promotes ILC2 development in the thymus (13). It has also been reported that RORα regulates Th2 cellular responses in allergic asthma. *Rora* deleted from CD4 T cells enhanced Th2 cellular responses, with increased IL-4/5/13–producing CD4 T cells following two models (*Aspergillus*/OVA and HDM) of allergen-induced inflammation and ex vivo stimulation (30). Therefore, RORα may have differential roles in regulating T cells, dependent on tissue and inflammation status. In human studies, there is an association of RORα with asthma (31–33), with *RORA* expressed in T cells of the airways of healthy and asthma patients (34). Furthermore, *RORA* expression was upregulated in patients with therapy-resistant asthma (35). However, the precise roles of RORα in the pathogenesis of asthma are not yet fully understood. To further examine the role of RORα in CD4 T cells during mouse models of allergic lung inflammation, we exposed mice to HDM pulmonary challenge. Consistent with the results observed in helminth-mediated lung inflammation, after HDM sensitization, there is an increase in the frequency of *Rora*-expressing Th2 cells and CD103 *Rora*-expressing CD4 T cells in the lungs. Furthermore, deletion of *Rora* from *CD4*-expressing cells resulted in a reduced frequency of Th2 cells in the lungs after HDM challenge. However, there was no difference in the HDM-induced pulmonary inflammation between *Rora^fl/fl^* and *Rora^fl/fl^CD4Cre* mice with comparable PAS score, BAL cell counts, eosinophilia, goblet cell hyperplasia, as well as levels of IL-4, IL-5, IL-17, and IFN-γ in lung tissue. These data indicate that although *Rora* has a role in Th2 cellular development during inflammation, *Rora*-expressing Th2 cells do not contribute to the genesis of lung inflammation after HDM treatment.

RORα is an important transcription factor in the development and function of several immune cells, including ILC2s, Th17 cells, Tregs, and macrophages. We identify a population of lung-resident *Rora*-expressing CD4 T cells, which expands during type 2 inflammation. We also demonstrate that full-functioning *Rora* is required for GATA3^+^CD4^+^ cellular development during pulmonary inflammation. Indeed, there is known plasticity in transcription factors that define CD4 cell lineages (36). Therefore, further studies are warranted to explore in more detail the mechanistic role for *Rora* in the lungs during GATA3^+^CD4^+^ Th2 cell development, including exploring other GATA3^+^CD4 cell lineages that coexpress other transcription factors, such as Foxp3^+^ Tregs. However, although we report *Rora* is required for the generation of Th2 cells in mice, *Rora*-expressing lung CD4 T cells have no functional role during *N. brasiliensis* infection and HDM challenge. These data demonstrate a new role for RORα in Th2 cellular development during pulmonary inflammation that could be relevant to the range of inflammatory diseases for which RORα is implicated.

## Supplementary Material

Supplemental 1 (PDF)
