# Supplementary material for: Retinoic Acid–Related Orphan Receptor α Is Required for Generation of Th2 Cells in Type 2 Pulmonary Inflammation
Source: J Immunol. 2023 Jun 30;211(4):626–32. doi: 10.4049/jimmunol.2200896 (PMC10404816; doi:10.4049/jimmunol.2200896)
Supplement: Supplemental 1 (PDF) [file JI_2200896_Supplemental_1.pdf]

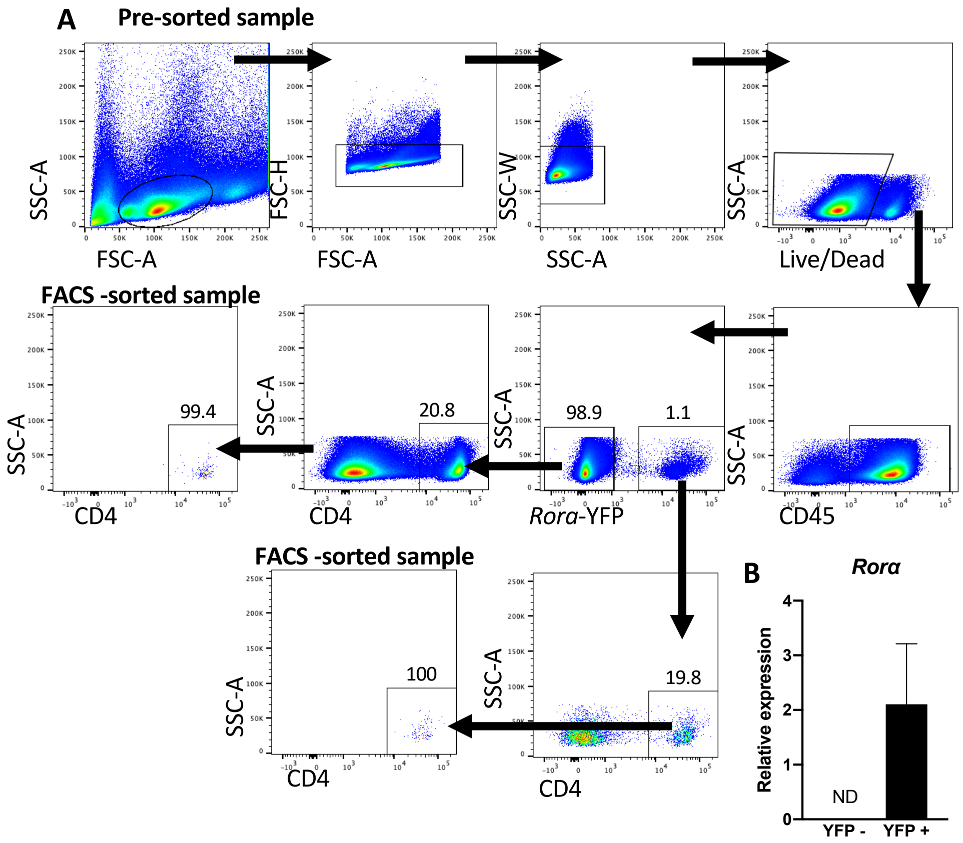


**Supplementary Figure 1**

**A**. Fluorescence-activated cell sorting of *Rora*-YFP$^+$ CD4 T cells. Splenocytes were prepared from *Rora*$^{Cre}$Rosa-YFP mice and stained with CD45 and CD4. The gating strategy excludes doublets and dead cells. The percentage of YFP$^+$ CD4 T cells is 19.8%. The resultant CD45$^+$CD4$^+$ cells are sorted based on YFP expression, into a YFP$^-$ and YFP$^+$ populations. The purity of the YFP$^+$ population was >99% and YFP$^-$ population was 100%. **B**. RNA was isolated from the sorted YFP$^+$ and YFP$^-$ CD4 T cells and expression of *Rora* quantified relative to 18S ($n$ = 2) ND = Non-detected.

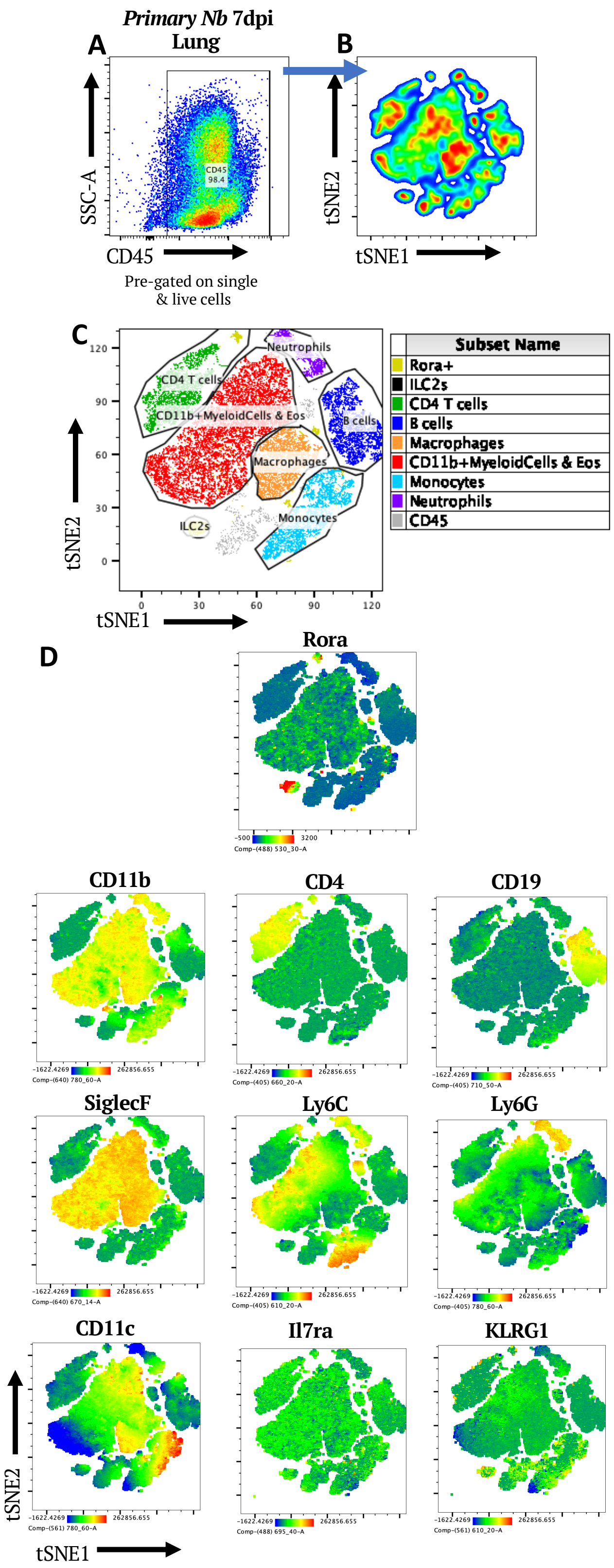


**Supplementary Figure 2**

**A.** CD45$^{+}$ cells were gated on single and live cells from lungs of *Rora*-YFP mice at day 7 following primary *N. brasiliensis* infection. **B.** CD45$^{+}$ cells were analysed by tSNE clustering and displayed as a pseudocolour density plot. **C.** Identification of lung immune cells (CD4 T cells, B cells, CD11b+ Myeloid Cells & eosinophils, macrophages, monocytes, ILC2s and neutrophils) and *Rora*-YFP expressing cells. **D.** tSNE clustering analysis of *Rora*-YFP, CD4, CD19, SiglecF, CD11b, Ly6C, Ly6G, CD11c, Il7ra and KLRG1. Eos = Eosinophils. Blue = lowly expressed. Red = highly expressed.

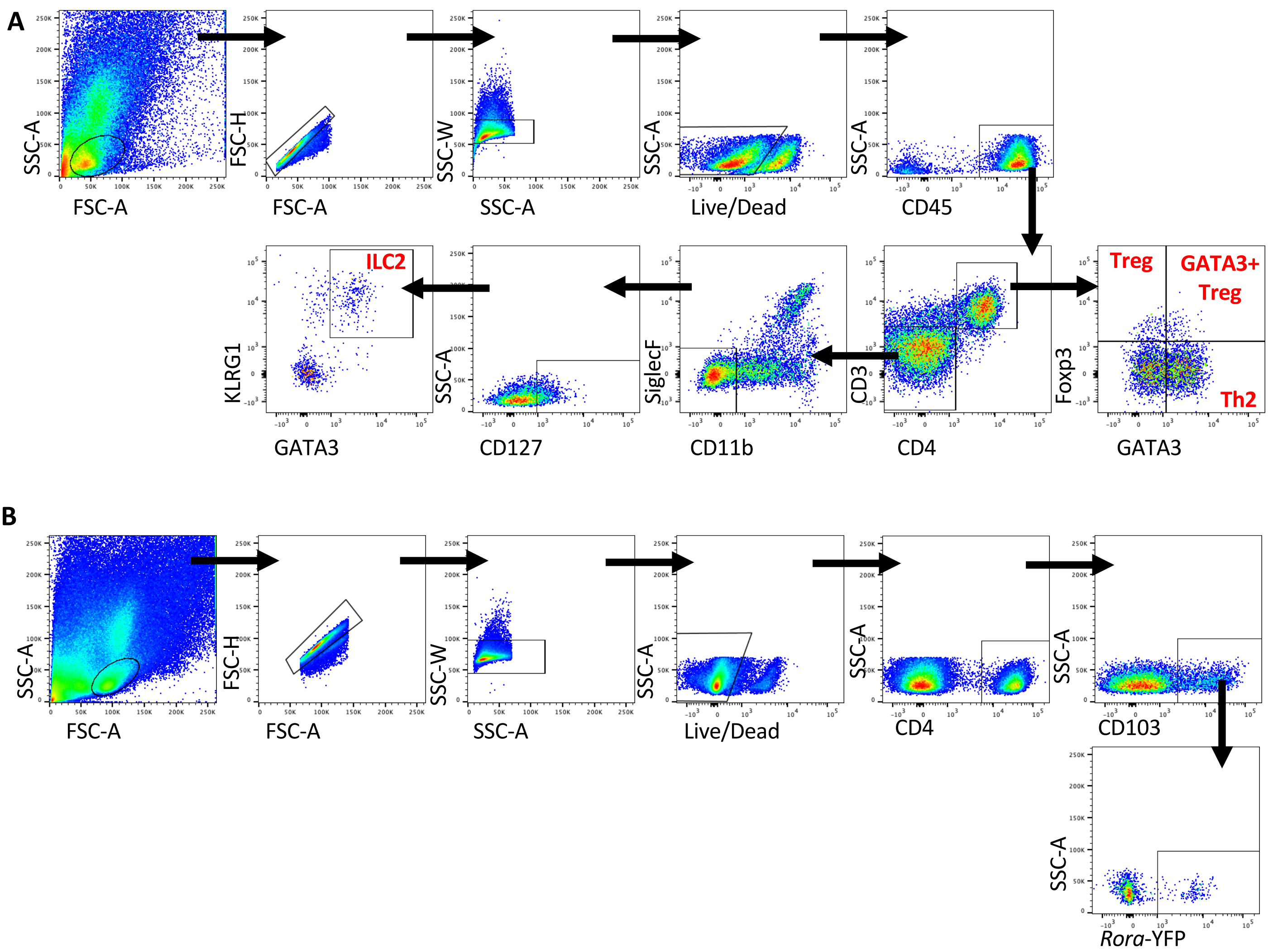


**Supplementary Figure 3**

**A.** Flow cytometry analysis of lung immune cells identifying ILC2s, Th2, Treg and GATA3$^{+}$Treg cells. Cells were gated as lymphocytes, singlets, Live/dead, CD45$^{+}$ and then CD4 T cells were assessed based on expression of Foxp3 (Treg), GATA3 (Th2) and GATA3$^{+}$Foxp3 (GATA3$^{+}$ Tregs). ILC2s were identified as CD4$^{-}$, CD3$^{-}$, CD11b$^{-}$, SiglecF$^{-}$, CD127$^{+}$, KLRG1$^{+}$ and GATA3$^{+}$. **B.** Flow cytometry analysis of lung immune cells identifying CD103$^{+}$ *Rora* expressing CD4 T cells. Cells were gated as lymphocytes, singlets, live/dead, CD4$^{+}$, CD103$^{+}$ and *Rora*-YFP$^{+}$.

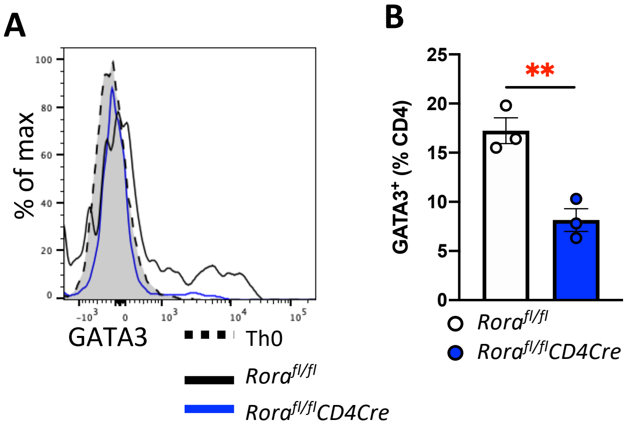


**Supplementary Figure 4**

**A.** Representative flow cytometry expression of GATA3 in CD4$^+$ T cells following *in vitro* Th2 cell polarisation. **B.** Frequency of GATA3$^+$ CD4 T cells following *in vitro* Th2 cell polarisation ($n$ = 3). Data is representative of mean ± SEM. Differences indicated as $p$ values, as assessed by Student $t$ Test. **$p$<0.01. ns = non-significant
